# Decellularized human amniotic membrane scaffolds: influence on the biological behavior of dental pulp stem cells

**DOI:** 10.1186/s12903-024-04130-y

**Published:** 2024-03-27

**Authors:** Zonghao Zhang, Bingling Lu, Luning Zou, Xinhui Huang, Fan Yang, Hongbing Lv

**Affiliations:** 1 Fujian Key Laboratory of Oral Diseases, Fujian Medical University, Fujian, 350000 China; 2 School and Hospital of Stomatology, Fujian Medical University, Fujian, 350000 China

**Keywords:** Human amniotic membrane, Biomaterials, Dental pulp stem cells, Extracellular matrix, Regenerative tissue engineering

## Abstract

**Objective:**

The objective of this study was to assess the characterization of human acellular amniotic membrane (HAAM) using various decellularization methods and their impact on the proliferation and differentiation of human dental pulp stem cells (DPSCs). The goal was to identify scaffold materials that are better suited for pulp regeneration.

**Methods:**

Six different decellularization methods were used to generate the amniotic membranes. The characteristics of these scaffolds were examined through hematoxylin and eosin (H&E) staining, scanning electron microscopy (SEM), and immunohistofluorescence staining (IHF). The DPSCs were isolated, cultured, and their capacity for multidirectional differentiation was verified. The third generation (P3) DPSCs, were then combined with HAAM to form the decellularized amniotic scaffold-dental pulp stem cell complex (HAAM-DPSCs complex). Subsequently, the osteogenic capacity of the HAAM-DPSCs complex was evaluated using CCK8 assay, live-dead cell staining, alizarin red and alkaline phosphatase staining, and real-time quantitative PCR (RT-PCR).

**Results:**

Out of the assessed decellularization methods, the freeze-thaw + DNase method and the use of ionic detergent (CHAPS) showed minimal changes in structure after decellularization, making it the most effective method. The HAAM-DPSCs complexes produced using this method demonstrated enhanced biological properties, as indicated by CCK8, alizarin red, alkaline phosphatase staining, and RT-PCR.

**Conclusion:**

The HAAM prepared using the freeze-thaw + DNase method and CHAPS methods exhibited improved surface characteristics and significantly enhanced the proliferation and differentiation capacity of DPSCs when applied to them. The findings, therefore demonstrate the capacity for enhanced pulp regeneration therapy.

**Supplementary Information:**

The online version contains supplementary material available at 10.1186/s12903-024-04130-y.

## Introduction

Endodontics and periapical diseases are prevalent oral conditions that are often characterized by inflammation or necrosis of the pulpal tissues. Historically, these conditions have been typically managed through root canal therapy. Nevertheless, following endodontic treatment, the tooth loses its ability to trigger an immune response, sense external mechanical and chemical stimuli, generate secondary dentin, and becomes more susceptible to longitudinal root fractures due to the removal of the inner wall of the root canal during the treatment procedure [1]. Fortunately, the emergence of oral tissue engineering has introduced novel approaches and methods for treatment [2].

The primary method employed for pulp regeneration in clinical settings is hemodialysis [3]. This method involves stimulating the apical foramen to induce bleeding, thereby creating regenerative ecological niches. Hemodialysis plays a crucial role in treating endodontics and periapical diseases in young permanent teeth. However, the regenerated tissues exhibit characteristics more akin to periodontal tissues rather than the pulp-dentin complex [4]. This distinction is likely because the stem cells responsible for regeneration originate from the periapical tissue rather than the pulp tissue. In recent times, there has been a growing interest in pulp regeneration strategies that involve the transplantation of stem cells. Accordingly, these methods have been successfully used in combination with scaffolds to regenerate the pulp-dentin complex [5, 6]; this makes pulp stem cells the most superior seed cell choice for pulp regeneration due to their distinct advantages. These include their ability to differentiate into neurovascular cells [7, 8], their immunomodulatory properties [9], and their ability to remain dry during cryopreservation [10]And it has a wide range of sources, low ethical controversy, easy availability, and low immunogenicity [11], making it the most superior seed cell choice for pulp regeneration.

Scaffolds play a crucial role in facilitating the colonization and proliferation of dental pulp stem cells (DPSCs) after transplantation. To ensure the optimal functioning of transplanted DPSCs, it is essential for the scaffolds to closely resemble the natural extracellular matrix (ECM) [12]. Human amniotic membrane (HAM) is a tissue that possesses a thickness ranging from 0.2 to 0.5 mm, making it the thickest basement membrane in the human body. It has a smooth surface and lacks blood vessels, nerves, and lymphatic vessels [13, 14]. Additionally, HAM contains ECM proteins, cytokines, and growth factors, which can enhance cell proliferation and provide antimicrobial properties [15]. However, when applied, the existence of cells causes the rejection of the graft. Previous efforts have been made to remove cells from other natural membrane structures, revealing the significant role of decellularized scaffolds in facilitating tissue regeneration [16, 17]. Thus, HAM underwent decellularization, a process that eliminates the cellular elements responsible for immune rejection, while preserving the structure, shape, and various cytokines of the ECM [18]. This results in a significantly reduced immunogenicity of the obtained HAAM [15]. Subsequently, the HAAM is utilized as a scaffold for tissues and as a carrier for delivering cells, drugs, or growth factors that aid in the promotion of healing and regeneration [19].

The methods employed for the preparation of decellularized amniotic membrane scaffolds (HAAM) typically encompass physical, chemical, and biological approaches. Physical methods such as freezing and thawing, mechanical oscillation, etc., achieve cell removal by disrupting cell membranes and causing cell lysis. These methods generally do not alter the composition and structure of the ECM. Nonetheless, a potential issue that may arise in the incomplete removal of cells [20]. On the other hand, chemical methods such as descalers, acid-base solutions, hypotonic and hypertonic solutions, etc. eliminate cells by rupturing cell membranes; however, they also damage protein components [21] and growth factors in the ECM [22, 23]. The use of biological methods, such as EDTA, nuclease, protease, etc., also allows for the separation of the cells [20]; however, they also interfere with the biologically active components of the ECM [24]. To summarize, while the impact of HAAM has been confirmed in various types of cells, there is still a lack of standardized decellularization methods. This presents a significant challenge for future clinical applications.

The present study, therefore, aimed to assess and compare the effectiveness of various decellularization methods on HAM, including the degradation of ECM. Additionally, we aimed to investigate the impact of HAAM prepared using different decellularization methods on the growth and bone-forming ability of dental pulp stem cells in a laboratory setting, to provide a basis for the in vitro study of the application of HAAM in dental pulp regeneration.

## Materials and methods

### Acquisition of human amniotic membrane tissue

This study was approved by the Ethics Committee of the Affiliated Stomatological Hospital of Fujian Medical University ([2019] Fu Medical Stomatology Ethics Review No. 32) and informed consent was obtained from all the participants. After obtaining written informed consent, our primary objective was to collect human amniotic membranes from medically fit women who underwent cesarean section at the 900 Hospital of the Joint Service Support Force of the People’s Liberation Army of China between January and March 2020. The eligibility criteria encompassed mothers within the age range of 25 to 35 years who had no recorded medical record of specific prenatal complications or infections, such as HBV, HCV, syphilis, and HIV.

### In vitro decellularization of amniotic membrane

Group A (AM): Fresh amniotic membranes were placed in a centrifuge tube and washed three times for 5 min each with a PBS solution containing 2% penicillin/streptomycin.

Group B (triton X-100) (Solarbio, China): The amniotic membrane was immersed in a centrifuge tube filled with a 1% triton X-100 solution. It was then agitated in a gas bath thermostat at 100 rpm for 24 h. Afterward, it was washed three times with a 2% PBS solution containing penicillin/streptomycin, with each rinse lasting 5 min.

Group C (trypsin + scraping) (Gibco BRL, America): The amniotic membrane was enzymatically digested in a 0.25% trypsin/EDTA solution at a temperature of 37 °C for 30 min. Subsequently, the epithelial cells of the amniotic membrane were carefully removed using a cell scraper, and the membrane was washed three times for 5 min each in a PBS solution containing 2% penicillin/streptomycin.

Group D (NaOH) (Sinopharm Chemical Reagent Co., Ltd., China): The amniotic membrane was immersed in a solution of 0.2% EDTA and incubated at 37 ℃ for 30 min. It was then washed in 0.5 M NaOH for 30 s, followed by rinsing in a solution of 5% NH_4_Cl under constant agitation. Finally, it was rinsed three times in a PBS solution containing 2% penicillin/streptomycin, with each rinse lasting 5 min.

Group E (EDTA) (Sinopharm Chemical Reagent Co., Ltd., China): The amniotic membrane was immersed in a 1% solution of EDTA at a temperature of 37 °C for 1 h. The amniotic membrane’s epithelial cells were manually extracted using a cell spatula, stacked individually, and subsequently washed three times for 5 min each in a PBS solution containing 2% penicillin/streptomycin.

Group F (freeze-thaw + DNase) (Gibco BRL, America): The amniotic membrane was submerged in a centrifuge tube containing a serum-free freezing solution. The tube was then placed in a refrigerator set at a temperature of -80 ℃ for 30 min, following which the membrane was frozen in liquid nitrogen for 30 min. Subsequently, the amniotic membrane was removed and heated in a water bath at 37 ℃ for 10 min, and the cycle was repeated three times. Next, the frozen-thawed amniotic membrane was immersed in 1 mg/ml of DNase enzyme, and the membrane was digested for 3 h at 37 ℃. Finally, the membrane was washed three times with a PBS solution containing 2% penicillin/streptomycin, with each wash lasting 5 min.

Group G (CHAPS) (Solarbio, China): The amniotic membrane underwent digestion using a 0.25% trypsin/EDTA solution at a temperature of 37 ℃ for a duration of 2 min. Subsequently, it was rinsed three times with a PBS solution containing 2% penicillin/streptomycin, with each rinse lasting 5 min. The membrane was then transferred to a decellularization solution consisting of a PBS solution containing 8 mM CHAPS, 25 mM EDTA, 0.12 M NaOH, and 1 M NaCl. It was incubated at room temperature with gentle agitation for a period of 7 h. Finally, the membrane was washed with a sterile solution. The specimen was placed in a PBS solution (comprising 8 mM CHAPS, 25 mM EDTA, 0.12 M NaOH, and 1 M NaCl) and incubated for 7 h at room temperature with mild stirring. Subsequently, it was washed three times with sterile distilled water for 15 min each time.

Group H (commercial HAAM) (JiangXi Ruiji BioTechnology Co., Ltd., China): The commercial biological amniotic membrane was placed in a centrifuge tube and washed with a PBS solution containing 2% double-antibody. This rinsing process was repeated three times, with each rinse lasting for 5 min.

### H&E staining

The H&E staining was conducted according to the standard protocol. Following deparaffinization and restoration of moisture, tissue sections were treated with a hematoxylin solution (ZSGB-BIO, China) for 5 min. Subsequently, the sections were immersed in a solution of 1% acidic ethanol (1% HCl dissolved in 75% ethanol) for a total of 5 cycles, and finally washed with distilled water. The sections were subsequently treated with eosin solution (ZSGB-BIO, China) for 3 min, followed by dehydration using alcohol of varying concentrations and clearing in xylene. The sections were subsequently examined and photographed using an Olympus BX53 microscope (Olympus, Japan).

### Hoechst nuclear staining

The amniotic membrane sections were incubated with Hoechst 33,342 (a dye that stains DNA, at a concentration of 10 µg/ml) for 10 min. Subsequently, fluorescence microscopy was employed to examine any remaining DNA present on the surface of the amniotic membrane.

### DNA quantitative analysis

The DNA assay kit was used in accordance with the instructions provided by the manufacturer. The DNA concentration of the diluted sample was measured at a wavelength of 260 nm, and the rate at which the amniotic membrane cleared the DNA was calculated.

### Immunohistofluorescence staining

Immunohistofluorescence staining was used to reveal the presence of important protein components (type I collagen, type III collagen, type IV collagen, type VI collagen, type VII collagen) in the amniotic membrane. The paraffin sections underwent antigen retrieval and were subsequently treated with primary antibodies, including anti-type I collagen, anti-type III collagen, anti-type IV collagen, anti-type V collagen, anti-type VI collagen, anti-type VII collagen, and anti-Laminin (1:200). These primary antibodies were incubated overnight at a temperature of 4 °C. Following this, the sections were treated with a secondary antibody, specifically IgG-FITC fluorescence, (1:100). The secondary antibody incubation took place in a wet box at a temperature of 37 °C for 1 h. For immunofluorescence staining, restaining was carried out using DAPI, and the images were captured for observation under a fluorescence microscope.

### SEM observation

The amniotic membrane from each group was dissected into small 3 mm × 3 mm pieces, with the corners cut to indicate the epithelial surface. The samples were then washed three times with PBS for 15 min each time. Subsequently, the samples were immersed in a 2.5% glutaraldehyde solution and fixed at room temperature for 2 h. Subsequently, the samples underwent two rounds of washing with PBS. Afterward, the samples underwent dehydration using an ethanol gradient (50, 70, 80, 90, 95, and 100%) for a duration of 10 min for each concentration. The freeze-dried decellularized amniotic membrane samples were then affixed to an adhesive tray using double-sided adhesive tape. Subsequently, they were examined under a scanning electron microscope to observe the surface structure of the different groups of amniotic membranes after being coated with gold using vacuum spraying.

### Cytotoxicity assessment

An equivalent amount of decellularized amniotic membrane was introduced into the α-MEM basal medium. The membrane was then sectioned using sterile ophthalmic scissors and soaked for 3 days. Afterward, it was subjected to centrifugation at 10,000 rpm, and the resulting supernatant was combined with 10% FBS to create the complete medium for the experimental group. The control group, on the other hand, received the complete medium with 10% FBS. Subsequently, the cell proliferation was assessed using the CCK8 method.

### Isolation and culture of Dental Pulp Stem cells (DPSCs)

The present study was reviewed by the Medical Ethics Committee of the Affiliated Stomatological Hospital of Fujian Medical University [(2016) Fu Medical Mouth Ethics Review No. (53)] and informed consent was obtained from all the participants. The inclusion criteria were as follows: Individuals with teeth that were free from dental decay, wisdom teeth that did not have periapical or periodontal lesions, or premolar teeth that had been extracted for orthodontic purposes. The teeth were thoroughly cleaned three times using a PBS solution that contained 2% double antibiotic. To disinfect the tooth surfaces, iodophor cotton balls were employed. The pulp was extracted by breaking open the teeth. Subsequently, a PBS solution containing 2% double antibiotic was used to clean the tooth three more times. Next, 1 mL of CD enzyme mixture, consisting of collagenase type I at a concentration of 3 mg/mL and dispase II enzyme at a concentration of 4 mg/mL, was added. The pulp tissues were then sheared using sterile ophthalmic scissors and placed in an incubator for digestion at a temperature of 37 °C and a CO_2_ concentration of 5% for 0.5–1 h. To stop the digestion process, 3 mL of complete medium (α-MEM containing 2% double antibody and 10% FBS) was added. The mixture was then centrifuged at 1000 rpm/min for 5 min, and the supernatant was discarded. The remaining pulp tissues were resuspended by adding 3 mL of complete medium. Subsequently, the suspension was inoculated into 25 cm^2^ flasks and supplemented with α-MEM complete medium containing 10% FBS and 1% double-antibody. The cells were incubated in a cell culture incubator at a temperature of 37 ℃ and a CO_2_ concentration of 5%. The cells were incubated in a cell culture incubator and the culture medium was replaced after 7 days. Once the cell growth reached 80% confluence, the cells were treated with trypsin at a ratio of 1:2–3 to detach them from the culture dish and transferred to a new dish. The 3rd to 5th generation of cells were used for subsequent experiments.

### Live and dead cell staining

The HAAM sample was divided into pieces measuring 1 cm × 1 cm. These pieces were then placed flat in a 24-well plate and secured using a cloning ring. Each well was supplemented with 1000 µL of α-MEM complete medium containing 10% FBS. The cells were then incubated at 37 ℃ and 5% CO_2_ for 30 min. After removing the old liquid, the cells were inoculated and placed back in the incubator for an additional 2–4 h at 37 ℃ and 5% CO_2_. Once the cells had adhered to the amniotic membrane, the plate was gently supplemented with 1 mL of complete medium and incubated for 5 days. Subsequently, 200 µL of live-dead dye was added to each well. Once the cells had firmly attached to the surface of the amniotic membrane, 1 mL of complete medium was carefully introduced into the culture plate, and the cells were incubated for 5 days. Subsequently, 200 µL of a solution containing live and dead dye was added to each well, and the cells were incubated at room temperature, shielded from light, for 20 min.

### CCK8 proliferative capacity assay

The cells were introduced into 96-well plates, each containing 5 sub-wells, and incubated at a temperature of 37 ℃ in a 5% CO_2_ incubator to promote cell adhesion to the plate surface. Subsequently, on the 1st, 3rd, 5th, and 7th day, a total of 10 µL of CCK8 solution mixed with 90 µL of α-MEM medium was added to each well at a specific time. Finally, the absorbance at 450 nm was then measured using an enzyme counter, and the average value for each group was used to create a line graph.

### Cell adhesion assay

The HAAM sample was divided into sections measuring 0.5 cm^2^ with a thickness of 2 mm and then placed on top of the DPSCs. Following a 4-hour incubation period at 37 °C and 5% CO_2_ in an incubator, the cells were enzymatically digested and the cell count was determined using a Countstar cell counting plate. The calculation was performed using the following formula.

$${\text{Adhesion}}\,{\text{rate}} = \frac{{{\text{Cell}}\,{\text{number}}\,{\text{of}}\,{\text{digested}}}}{{{\text{Cell}}\,{\text{number}}\,{\text{of}}\,{\text{seeded}}}} \times 100$$

### Alizarin red and alkaline phosphatase staining

The groups were placed in 24-well plates and exposed to osteogenic induction using a solution containing α-MEM medium, 10 nmol/L dexamethasone, 10 mmol/L sodium β-glycerophosphate, 50 µg/ml vitamin C, and 100 ml/L FBS. The staining procedures were as follows: During the 21-day Alizarin Red Staining procedure, the culture medium was first removed from the wells. Then, the wells were washed with PBS and subsequently fixed in a 4% paraformaldehyde solution for 30 min. Following that, three PBS washes were performed, and the sample was then stained with a 1% pH 4.1 alizarin red solution for 10 min. The wells were then rinsed with distilled water to prepare them for microscopic observation, which aimed to evaluate the formation of calcified nodules. In addition, the 7-day alkaline phosphatase staining involved treating the wells with BCIP/NBT staining solution for 30 min at room temperature, while ensuring protection from light. Following two rinses with distilled water, the samples were observed using an inverted microscope.

### RT-PCR analysis of osteogenesis-related gene expression

The expression of the osteogenic differentiation-related genes in DPSCs was assessed using quantitative RT-PCR (qRT-PCR). Herein, after being exposed to osteogenic induction for a period of 14 days, the cells had their total RNA extracted using TRIzol (Invitrogen). The PrimeScript™ RT kit (TaKaRa, RR047A) was used for the subsequent cDNA synthesis. The primers for the target gene are detailed in Table S1. The amplification was carried out using the SYBR Premix Ex Taq II kit (TaKaRa, RR820A). Additionally, the 2-ΔΔCt method was used to standardize the expression of the target gene relative to the levels of GAPDH.

**Table:** 

Genes	Primer sequences
GAPDH Forward	5’-CAGGAGGCATTGCTGATGAT-3’
GAPDH Reverse	5’-GAAGGCTGGGGGCTCATTT-3’
ALP Forward	5’-CTGGACCTCGTTGACACCTG-3’
ALP Reverse	5’-TCCGTCACGTTGTTCCTGTT-3’
COL1A1 Forward	5’-GAGGGCCAAGACGAAGACATC-3’
COL1A1 Reverse	5’-CAGATCACGTCATCGCACAAC-3’
OCN Forward	5’-CTCACACTCCTCGCCCTAT-3’
OCN Reverse	5’-TCTCTTCACTACCTCGCTGC-3’

### Statistical analysis

Each trial was replicated thrice under identical conditions. The resultant data were analyzed using the SPSS 20.0 software, and the results were presented as mean ± standard deviation. In addition, the statistical significance of the results was assessed using the One-Way ANOVA test, where a P-value < 0.05 indicated significance.

## Results

### Characterization of HAAM

#### Gross morphology of amniotic membrane

**Fig. 1:**
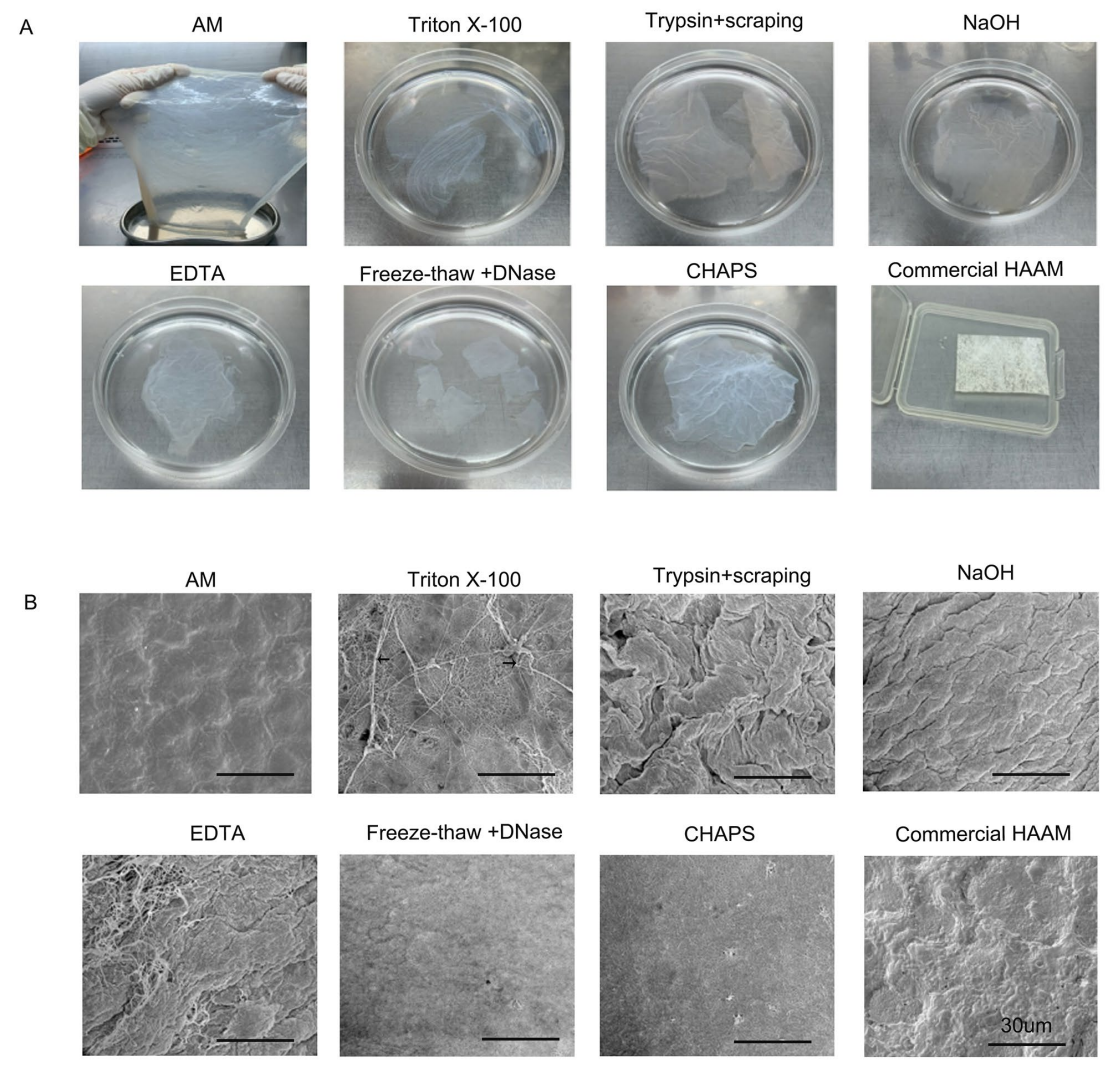
Gross view and microscopic structure (SEM) of the decellularized amniotic membrane. **(A)** Gross morphologies of the primitive amniotic membrane and the decellularized amniotic membrane prepared using different decellularization methods. **(B)** Microscopic structures (SEM) of the primitive amniotic membrane and the decellularized amniotic membrane prepared using different decellularization methods.Black arrow indicates fibre release

The gross view of the amniotic membrane (Fig. 1A) revealed a fresh, white, or yellow appearance. In addition, it appeared translucent, lacking blood vessels and nerves, and possessed a smooth texture, exhibiting strong elasticity. Following decellularization, the HAAM in each group exhibited a white or creamy white appearance, with a smooth texture. However, there was a decrease in elasticity, thickness, and an increase in permeability, while the HAAM in each group remained structurally intact. In addition, the triton X-100 group and trypsin + cellular scraping group were found to have the least compact texture, while the CHAPS group exhibited the highest level of permeability. The commercial amniotic membrane group appeared as a white film with strong elasticity and a concave and convex surface.

#### SEM analysis

The native amniotic membrane exhibited a level surface with epithelial cells organized in a compact manner, as evident from the SEM analysis. However, following decellularization, certain membranes displayed wrinkling. The trypsin + cell scraping and NaOH groups, in particular, exhibited significant folding. In addition, evidence of collagen fiber damage was observed in groups treated with tritonX-100. Conversely, the membranes obtained from the freeze-thaw + DNase and CHAPS groups displayed intact surfaces characterized by interwoven fibers (Fig. 1B).

### HAM decellularization evaluation

#### Post-decellularization matrix analysis

**Fig. 2:**
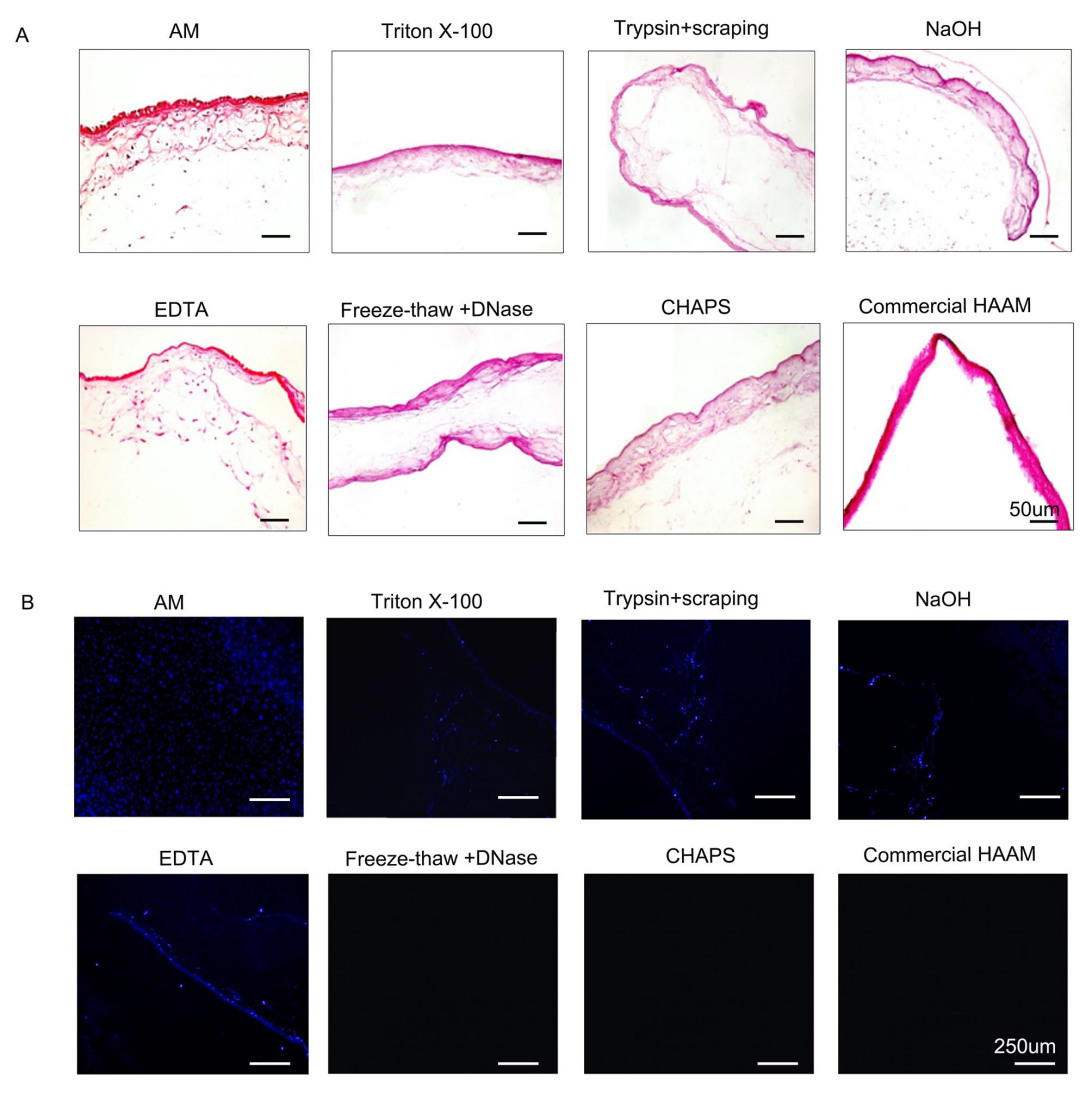
H&E staining and Hoechst staining of HAAM **(A)** H&E of HAAM treated by different methods **(B)** Hoechst staining of HAAM treated by different methods

As observed in the H&E staining, the intact membranes exhibited a uniform epithelial layer with clearly visible blue-purple nuclei. Additionally, a prominent basement membrane and a lax stroma layer were also detected. However, following decellularization, the membranes from specific groups, particularly those treated with trypsin and cell scraping, as well as NaOH, exhibited irregularities in the basement membrane. In addition, the EDTA-treated group showed the presence of residual cells, while the freeze-thaw + DNase and CHAPS groups displayed relatively intact layers with a thickness comparable to that of the amniotic membrane. The commercial group also exhibited a deeply stained stroma (Fig. 2A).

#### Analysis of nuclear staining after decellularization

The cell nuclei exhibited a blue color when observed under fluorescence. In this context, the AM group demonstrated a substantial amount of blue fluorescence, followed by the EDTA group. Conversely, only a minimal level of blue fluorescence was observed in the triton X-100, trypsin + scraping, and NaOH groups, while the rest of the groups did not exhibit any blue fluorescence. This indicates that the freeze-thaw + DNase group, CHAPS group, and commercial HAAM group achieved a highly effective decellularization effect (Fig. 2B).

#### DNA content analysis

Following decellularization, a significant reduction in the amount of DNA was observed. In this context, the freeze-thaw + DNase (86.26 ± 1.1%) and CHAPS (82.94 ± 2.4) groups exhibited the most effective DNA removal rates (Table S1, Fig. S1).

### Analysis of collagen retention after decellularization

**Fig. 3:**
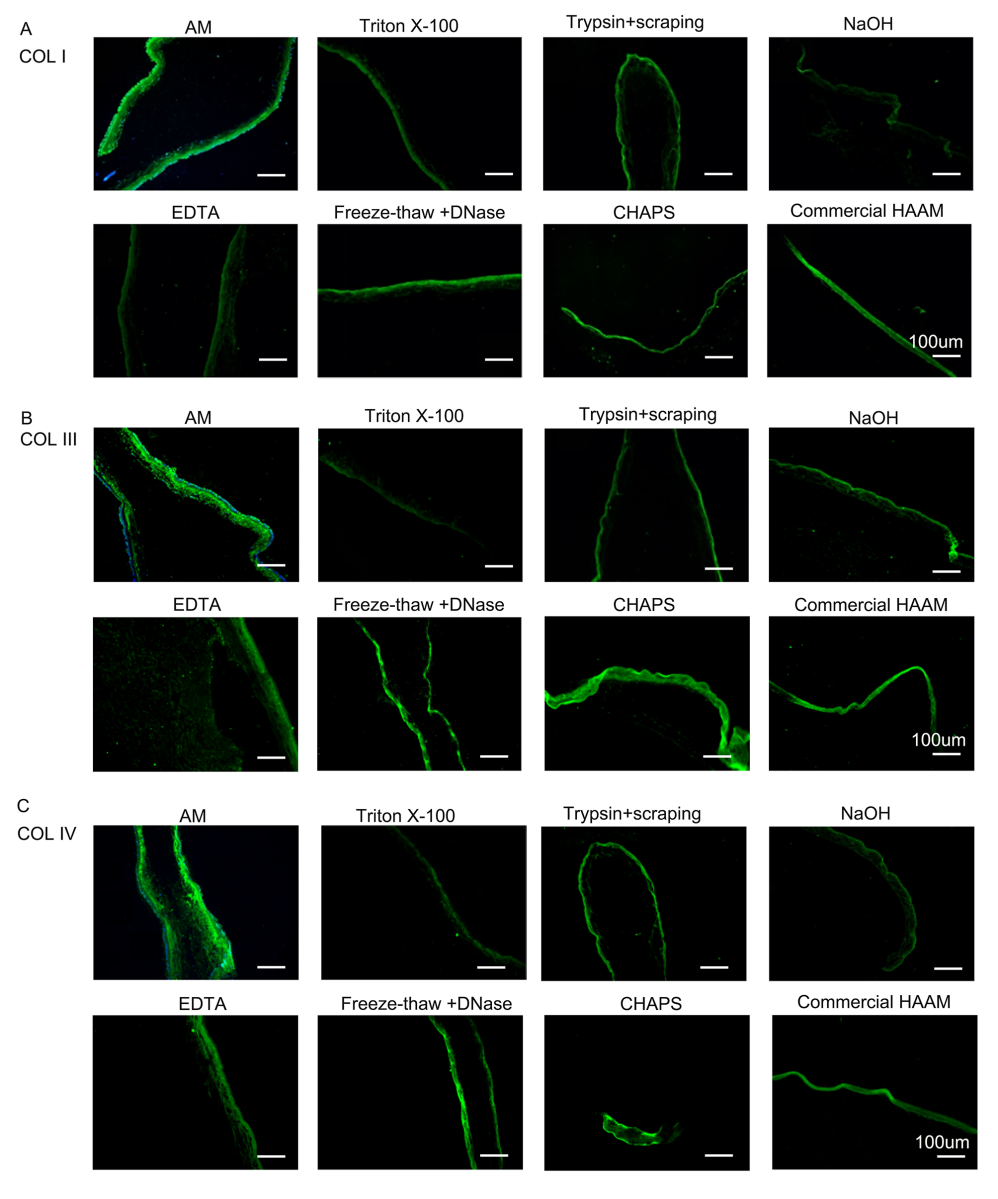
IHF of HAAM (COL I, COL III, COL IV). **(A)** IHF of COL I of HAAM treated by different methods **(B)** IHF of COL III of HAAM treated by different methods **(C)** IHF of COL IV of HAAM treated by different methods

**Fig. 4:**
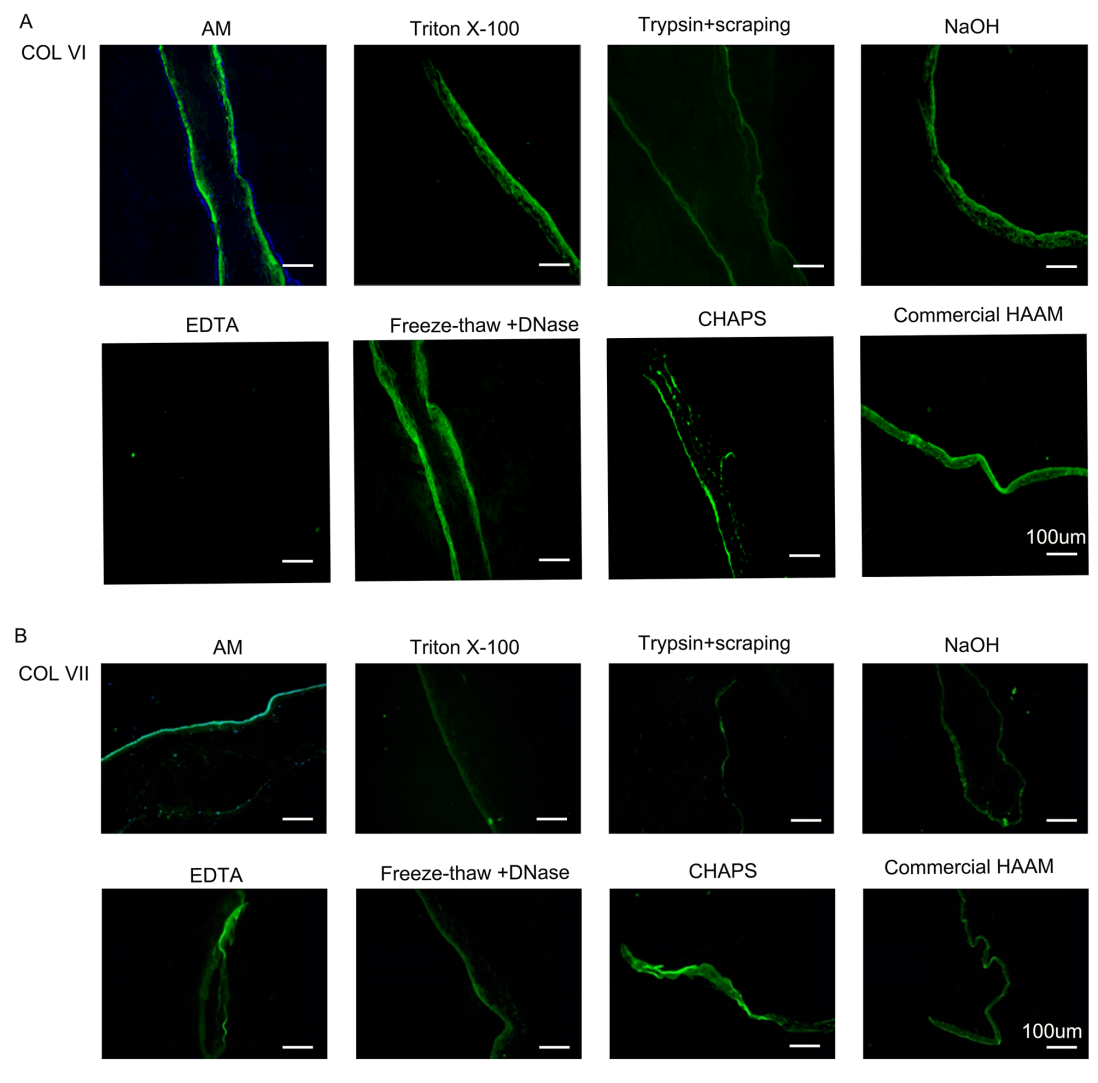
IHF of HAAM (COL VI, COL VII). **(A)** IHF of COL VI of HAAM treated by different methods **(B)** IHF of COL VII of HAAM treated by different methods

The AM group exhibited robust positivity for all collagen types. Furthermore, following decellularization, the COL (I, III, IV), trypsin + cell scraping, freeze-thaw + DNase, CHAPS, and commercial HAAM groups demonstrated increased collagen expression, whereas the remaining groups exhibited a notable decrease. Among them, the triton X-100 group displayed the most substantial decrease. In the case of COL VI, the triton X-100, freeze-thaw + DNase, CHAPS, and commercial HAAM groups exhibited elevated expression levels, whereas the remaining groups displayed significantly reduced expression levels, with the EDTA group demonstrating virtually no expression. In COL VII, the groups treated with EDTA, triton X-100, freeze-thaw + DNase, CHAPS, and commercial HAAM showed higher expression levels. Conversely, the other groups exhibited significantly lower expression levels, and the trypsin + scraping group showed almost no expression (Figs. 3 and 4).

### Toxicity analysis of HAAM extract

The DPSCs proliferation assays conducted using HAAM extracts demonstrated accelerated growth rates in comparison to the control groups. Notably, the CHAPS and freeze-thaw + DNase groups exhibited the highest levels of proliferation (Fig. S2).

### Effect of HAAM on DPSC proliferation and activity

The isolation and characterization of DPSCs were achieved by successfully through immunomagnetic bead sorting. The cells, which were derived from the mesoderm and possessed the capacity to differentiate in multiple directions, were used as the initial cells for further investigations (Fig S3).

**Fig. 5:**
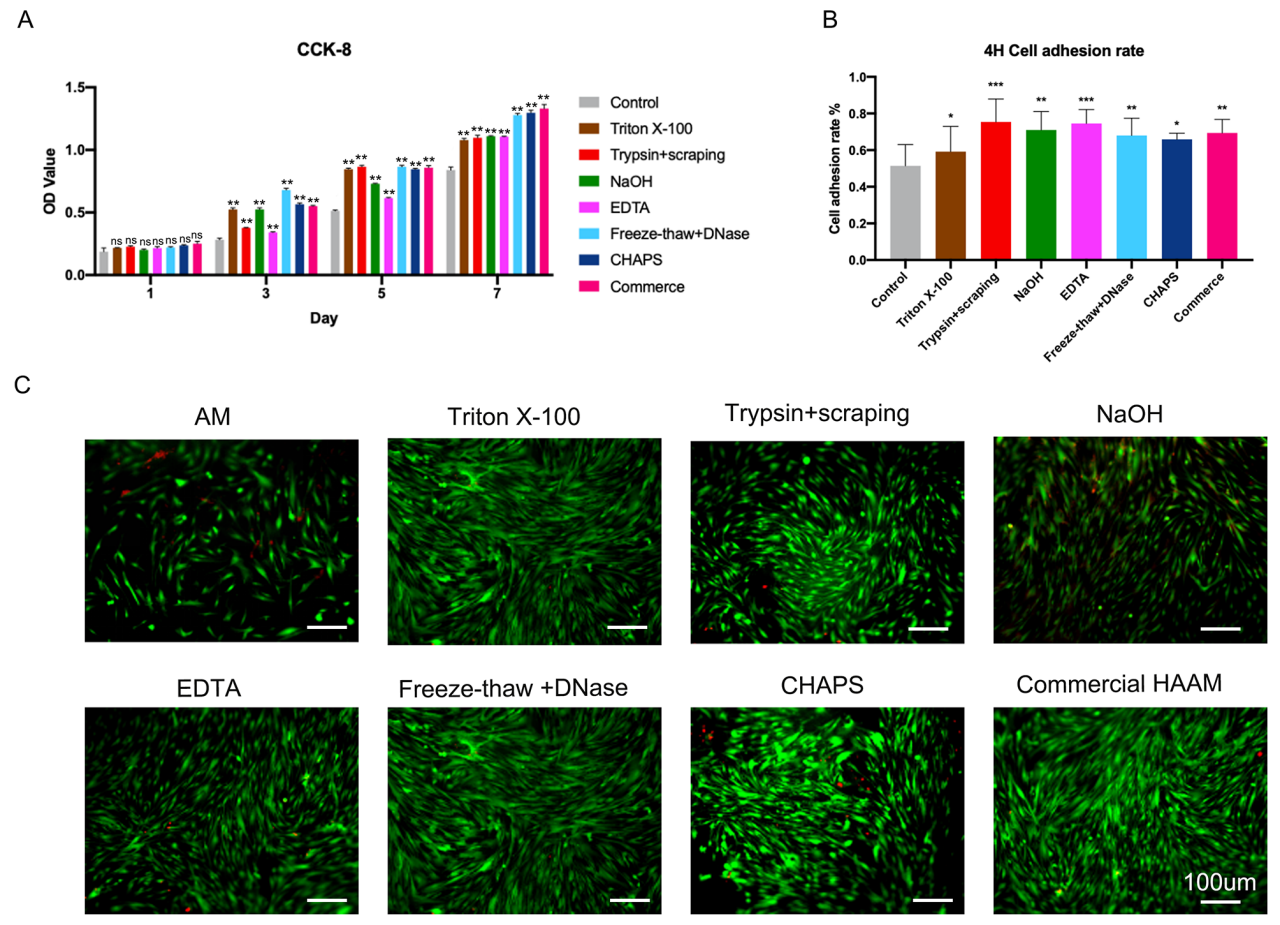
Effect of HAAM on the proliferation of DPSCs, adhesion ability, and fluorescence staining of live dead cells. **(A)** Effect of HAAM on DPSC proliferation. **(B)** Effect of HAAM on the adhesion ability of DPSCs. **(C)** Fluorescence staining of live and dead cells of HAAM-DPSC complexes treated using different methods.*: significant difference (*P* < 0.05), **: highly significant difference (*P* < 0.01), ***: highly significant difference (*P* < 0.001)

After analyzing the ability of the HAAM-DPSC complex to multiply, it was evident that all groups treated with HAAM-DPSC showed a significant increase in proliferation compared to the group treated with only cell plate (*P* < 0.01). Among these, the freeze-thaw + DNase, CHAPS, and commercial amniotic membrane groups exhibited the most significant proliferation following a 7-day observation (Fig. 5A).

Furthermore, the adhesive capacity of the HAAM-DPSC complex was assessed. The results of the 4-hour adhesion rate assay showed that the HAAM-DPSC complex groups had significantly higher rates of adhesion compared to the cell plate group (*P* < 0.05) (Fig. 5B). Additionally, the results of the live-dead cell staining further revealed that only the NaOH group exhibited a lower number of live cells compared to the cell plate group. The fluorescence intensity was also enhanced in other groups; however, the CHAPS group demonstrated the most significant increase (Fig. 5C).

### Effect of HAAM on DPSC osteogenic differentiation

**Fig. 6:**
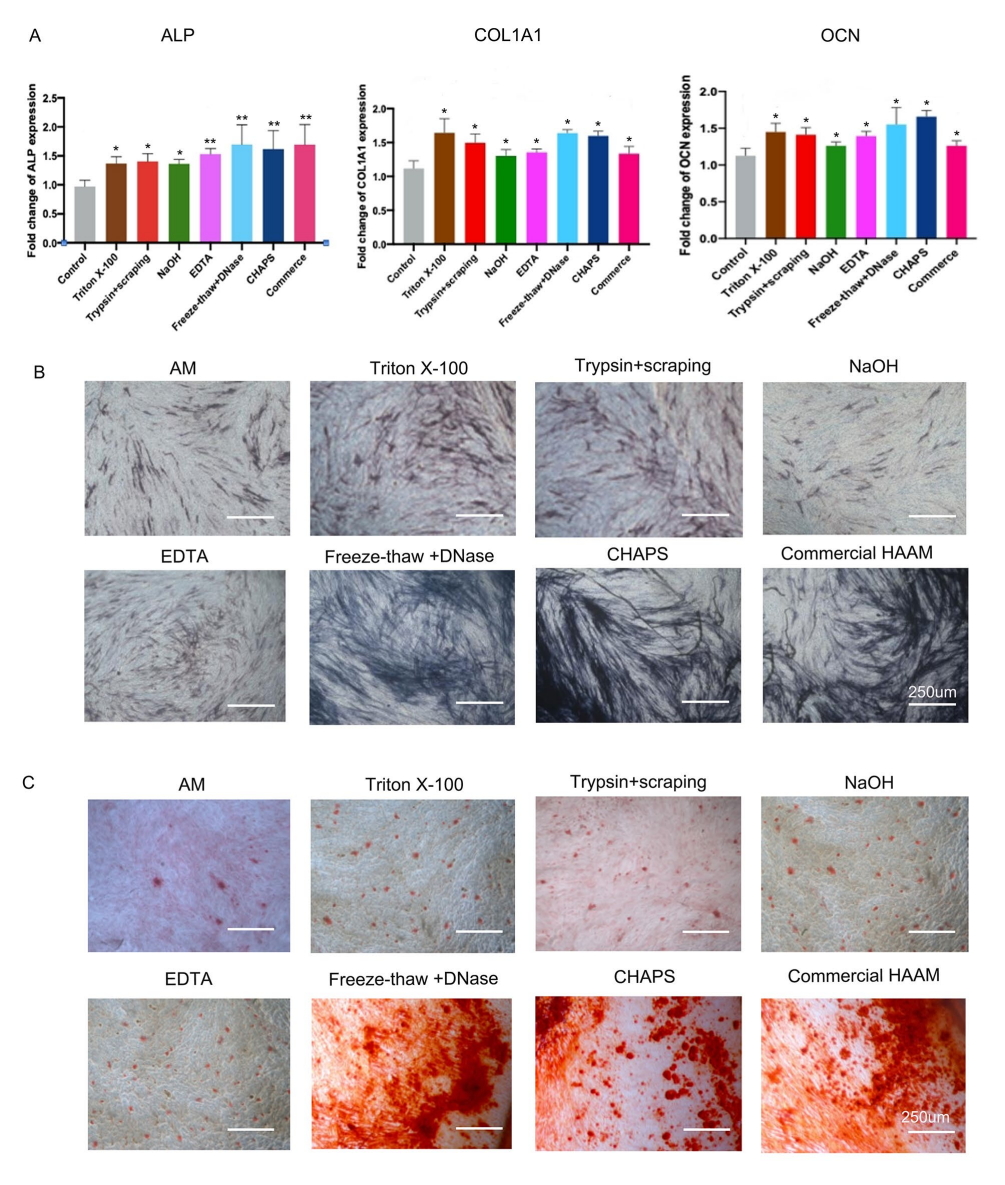
Expression of the osteogenesis-related genes in the HAAM-DPSC complex and staining with alizarin red and alkaline phosphatase. **(A)** RT-PCR to detect the expression of osteogenesis-related genes. **(B)** Alkaline phosphatase staining of the HAAM-DPSC complex. **(C)** Alizarin red staining of the HAAM-DPSC complex.*: significant difference (*P* < 0.05), **: highly significant difference (*P* < 0.01), ***: highly significant difference (*P* < 0.001)

After inducing osteogenic differentiation in the HAAM-DPSC complex, an analysis using RT-PCR was conducted to examine the expression levels of osteogenesis-related genes (ALP, COL1A1, and OCN) after 14 days. The results showed that the HAAM-DPSC complex exhibited higher expression levels of these genes compared to the control group (Fig. 6A). In particular, the groups that underwent freeze-thaw + DNase treatment, CHAPS treatment, and commercial amniotic membrane treatment, demonstrated a notable increase in the ALP expression, when compared to the other groups.

Following ALP staining after 7 days (Fig. 6B), there was an increase in the intensity of staining observed in the HAAM-DPSC complex in all groups compared to the cell plate group. In particular, the freeze-thaw + DNase, CHAPS, and commercial amniotic membrane groups showed a significant increase in staining. Correspondingly, on the 21st day, alizarin red staining (Fig. 6C) revealed that the HAAM-DPSC complex groups had a higher number of calcium nodules compared to the cell plate group. Notably, the freeze-thaw + DNase, CHAPS, and commercial amniotic membrane groups exhibited the highest increase in calcium nodule formation, indicating that these decellularization methods effectively enhance DPSC osteogenic differentiation, surpassing other methods.

## Discussion

The objective of the present study was to evaluate different decellularization methods used in previous studies to identify the most appropriate method for the generation of HAAMs. This would establish a standardized approach for HAAM preparation in future clinical applications. Furthermore, to investigate the potential application of HAAM-DPSCs complexes in pulp regeneration therapy, we aimed to conduct in vitro experiments involving the piggybacking of DPSCs.

Herein, triton X-100 [25], trypsin + cell scraping [26], NaOH [27], EDTA [28], freeze-thaw + DNase [29], and CHAPS [30] were utilized to generate HAAMs. As observed, all decellularization methods successfully preserved the structural integrity of the HAAM without causing lysis or fragmentation. In addition, the results of the quantitative DNA analysis revealed that the decellularized amniotic membrane group had DNA residues below 50 ng/mg. Retaining a matrix DNA concentration below 50 ng/mg is deemed essential to avoid exacerbating the inflammatory response after implantation [31]. As observed from the findings, all DNA residues in the decellularized amniotic membranes were found to be below 50 ng/mg. Furthermore, all of the aforementioned decellularization methods effectively eliminated epithelial and stromal cells. However, the freeze-thaw + DNase group and the CHAPS group exhibited the highest efficiency in cell removal. In addition, these two groups demonstrated the most effective cell removal among all the methods mentioned. On the other hand, the DNase and CHAPS groups were found to retain a significant quantity of collagen. Collagen retention is essential for the integrity of HAAM, which comprises a particularly important structure in the amniotic matrix. Type I collagen is an extracellular matrix protein that plays associated crucial role in the growth and maturation of bone tissue and stimulates the adhesion and differentiation of osteoblasts [32]. Earlier studies have shown that Type I collagen creates a favorable environment that directs the transformation of stem cells into osteoblasts [33]. On the other hand, Type III collagen is primarily released during periosteal regeneration and facilitates the repair of bone tissue. In this context, Joerring et al., discovered that following a tibial fracture, the area of injury exhibited an elevated concentration of type III collagen compared to the unaffected area [34]. Conversely, the remaining members of the group exhibited varying levels of collagen deficiency, which aligns with previous research findings [21–23].

Although the methods mentioned above for decellularization are effective, they do not currently allow for the complete removal of cells. This, in turn, may result in collagen destruction if excessive removal of cells occurs. Thus, the selection of seed cells that have an immune-modulating effect to prevent potential immune rejection is particularly important. DPSCs possess a unique characteristic of neurovascular differentiation, indicating that they are highly suitable candidates for the regeneration of dental pulp tissues [7, 35, 36]. In addition, DPSCs facilitate the angiogenic process through the secretion of pro-angiogenic factors and their direct differentiation into perivascular and endothelial cells [37–39]. Angiogenesis facilitates the provision of nutrients, oxygen, and minerals, which are crucial for mineralization and the creation of bone [40, 41]. Additionally, DPSCs possess strong immunomodulatory capabilities that help to alleviate immune rejection [42, 43]. The co-culture of activated T cells with DPSCs suppresses T cell proliferation by inducing the generation of regulatory T cells (Tregs). This indicates that Tregs may have a significant impact on the immunosuppressive characteristics of DPSCs [44, 45]. Additionally, DPSCs are stimulated to secrete IFN-γ, which triggers the release of TGF-β from DPSCs, resulting in their immunosuppressive effects [42].

Previous studies have demonstrated that HAAM can stimulate the growth and movement of DPSCs [46], which aligns with our findings. However, investigations elucidating the correlation between HAAM and the differentiation capacity of DPSCs have been very limited. Herein, we piggybacked DPSCs onto HAAM and it was observed that HAAM prepared in all groups could promote the osteogenic differentiation of cells. However, the freeze-thaw + DNase group, as well as the CHAPS group exhibited the highest osteogenic ability. This is in line with the results of the immunofluorescent staining of collagen in the previous period and further supports the effectiveness of the scaffolds. The importance of collagen, the manifestation of genes related to osteogenic differentiation such as ALP, COL1, OCN, and alkaline phosphatase, and the outcomes of alizarin red staining also substantiate this, thereby indicating that while HAAM can enhance the growth, differentiation, and other biological activities of DPSCs, variations still arise due to different decellularization methods. The present study demonstrated that the employment of the freeze-thaw + DNase and the CHAPS method for preparing HAAM is significantly superior in terms of amniotic membrane decellularization. Furthermore, it promotes the proliferation and osteogenic differentiation of DPSCs more effectively.

## Conclusions

The present study identified the two most optimal decellularization methods, viz., the freeze-thaw + DNase method and the CHAPS method for the generation of HAAMs. In addition, it demonstrated the efficacy of these methods in enhancing the regenerative capabilities of DPSCs. The resulting findings, thus, suggest that these methods could be a superior option for delivering DPSCs, thereby laying the groundwork for future in vivo experiments and clinical trials focused on dental pulp regeneration.

### Electronic supplementary material

Below is the link to the electronic supplementary material.

Supplementary Material 1

Supplementary Material 2

Supplementary Material 3

Supplementary Material 4

## Data Availability

All data generated or analyzed during this study are included in this published article.
