# Supplementary material for: Decellularized human amniotic membrane scaffolds: influence on the biological behavior of dental pulp stem cells
Source: BMC Oral Health. 2024 Mar 27;24:394. doi: 10.1186/s12903-024-04130-y (PMC10976669; doi:10.1186/s12903-024-04130-y)
Supplement: Supplementary file 1 — Supplementary Material 1 [file 12903_2024_4130_MOESM1_ESM.docx]

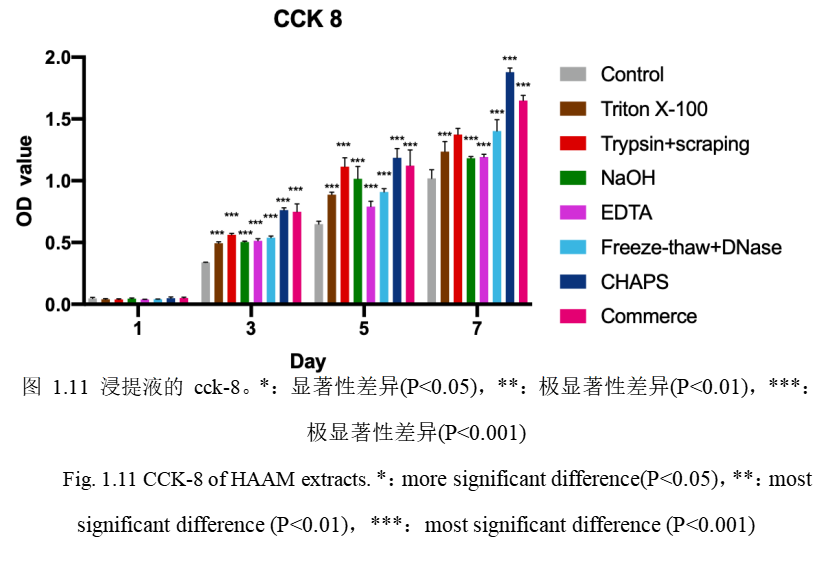


Fig S2. cck-8 of different HAAM extracts. *: significant difference (P<0.05), **: highly significant difference (P<0.01), ***: highly significant difference (P<0.001)
