# Supplementary figures and images for: Decellularized human amniotic membrane scaffolds: influence on the biological behavior of dental pulp stem cells
Source: BMC Oral Health. 2024 Mar 27;24:394. doi: 10.1186/s12903-024-04130-y (PMC10976669; doi:10.1186/s12903-024-04130-y)

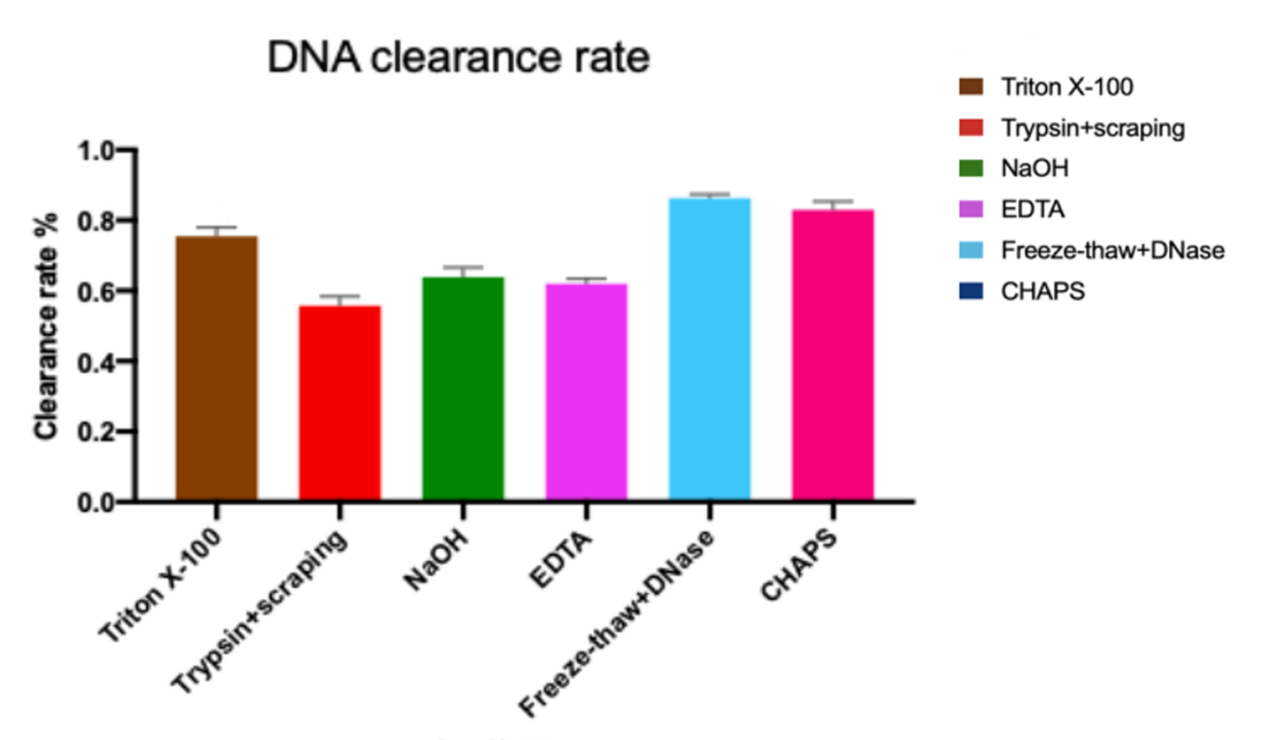


1. Fig S1. DNA clearance rate

Supplement: Supplementary file 2 — Supplementary Material 2 [file 12903_2024_4130_MOESM2_ESM.docx]
