# Supplementary material for: Decellularized human amniotic membrane scaffolds: influence on the biological behavior of dental pulp stem cells
Source: BMC Oral Health. 2024 Mar 27;24:394. doi: 10.1186/s12903-024-04130-y (PMC10976669; doi:10.1186/s12903-024-04130-y)
Supplement: Supplementary file 3 — Supplementary Material 3 [file 12903_2024_4130_MOESM3_ESM.docx]

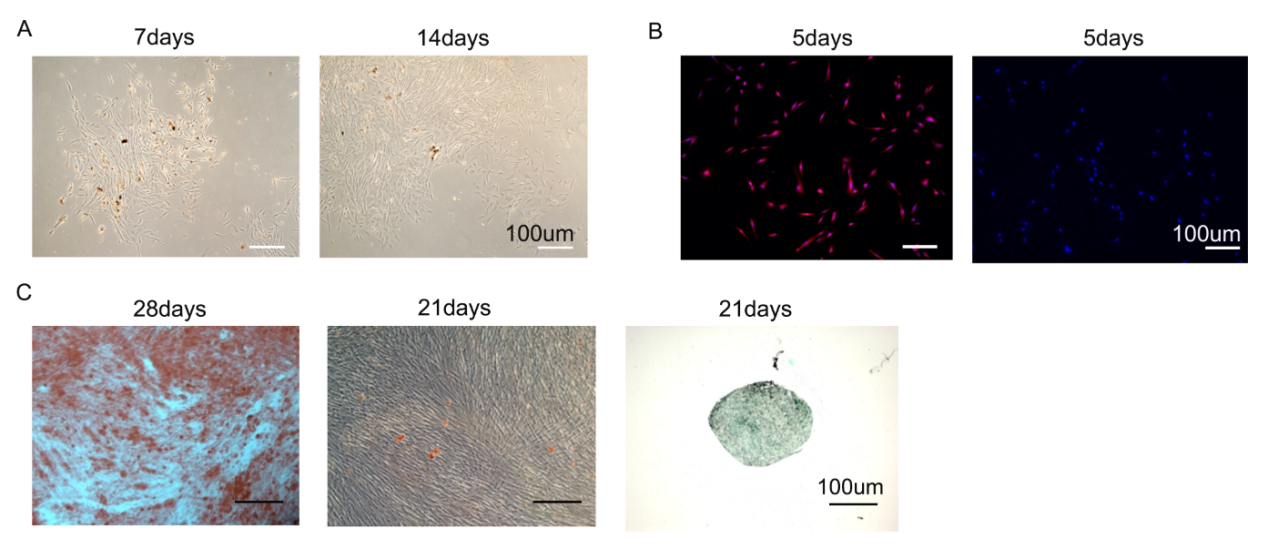


Fig S3. stemness identification of dental pulp stem cells (A) Microscopic morphology of dental pulp stem cells (B) Immunofluorescence of anti-polymorphin and anti-keratin of DPSC (C) Staining for osteogenic, lipogenic and chondrogenic differentiation of DPSC at day 21.
