# Supplementary material for: Decellularized human amniotic membrane scaffolds: influence on the biological behavior of dental pulp stem cells
Source: BMC Oral Health. 2024 Mar 27;24:394. doi: 10.1186/s12903-024-04130-y (PMC10976669; doi:10.1186/s12903-024-04130-y)
Supplement: Supplementary file 4 — Supplementary Material 4 [file 12903_2024_4130_MOESM4_ESM.docx]

| clusters | DNA removal rate/100% |
| --- | --- |
| Triton X-100 | 75.48±2.5 |
| Trypsin + cell scraping | 55.74±2.6 |
| NaOH | 63.89±2.7 |
| EDTA | 61.98±1.4 |
| freeze-thaw + DNase | 86.26±1.1 |
| CHAPS | 82.94±2.4 |

Table S1.DNA clearance rate of decellularized amniotic membrane (x±s)
